# Characteristics of rice straw decomposition and bacterial community succession for 2 consecutive years in a paddy field in southeastern China

**DOI:** 10.1038/s41598-022-25229-8

**Published:** 2022-12-03

**Authors:** Xiya Wang, Ping He, Xinpeng Xu, Shaojun Qiu, Shicheng Zhao

**Affiliations:** Key Laboratory of Plant Nutrition and Fertilizer, Ministry of Agriculture and Rural Affairs, Institute of Agricultural Resources and Regional Planning, Chinese Academy of Agricultural Sciences, Beijing, 100081 China

**Keywords:** Biochemistry, Microbiology

## Abstract

The characteristics of long-term rice straw decomposition and succession in the bacterial community in the double-rice system are still unclear. Here a 2-year continuous straw bag decomposition experiment was conducted to explore changes in nutrient release, enzyme activity, and bacterial community composition during rice straw decomposition in the double-rice system in Southeast China. After burial in soil, the cumulative dry matter loss rates of rice straw were 38.9%, 72.6%, and 82.7% after 2, 12, and 24 months, respectively. The change in the release rate of straw nitrogen and phosphorus was similar to the dry matter loss, but 93.5% of straw potassium was released after 1st month. Bacterial abundance and community diversity in straw increased rapidly, reaching peaks after 7 and 12 months, respectively. Straw extracellular enzyme activities were the highest in the first 2 months and then gradually decreased over time, and they significantly and positively correlated with straw decomposition rate. Straw decomposition was dominated by copiotrophic *Bacilli* and *Flavobacteriia* in the early stages and evolved to be dominated by oligotrophic *Acidobacteria*, *Anaerolineae*, *Deltaproteobacteria*, *Saccharibacteria*, and *Sphingobacteriia* in the later stages. Changes in the C/N and K content of straw are the main reasons for bacterial community succession during rice straw decomposition. This study can provide a scientific basis for developing efficient decomposing bacteria agents for rice straw.

## Introduction

Crop straw, as an organic by-product of agricultural production, contains rich nutrients needed for plant growth. It has been estimated that 31.6%, 22.5% and 28.8% of the amount of nitrogen required for rice, wheat and corn production can be supplied by returning all the corresponding crop straw to the field in China^1^. The amount of potassium that can be supplied by returning rice, wheat and corn straw to the field in the current season is 4.99, 1.93 and 4.79 million tons, respectively^2^. After crop straws are returned to the soil, their mineral nutrients are gradually released during the decomposition process, and the process is mainly regulated by soil microbes. Straw quality and other environmental conditions, such as soil water and temperature, also affect the decomposition process by influencing the abundance and community composition of soil microbes^3–5^. Therefore, exploring the nutrient release pattern during straw decomposition can guide the fertilization measures after returning rice straw to field.

Microorganisms and enzymes play an important role in straw decomposition. Previous studies showed that the rapid decomposition of soluble organic components such as soluble sugars, amino acids and proteins, as well as hemicellulose and cellulose, occurs within the straw due to soil microbial activity in the early stages of straw decomposition^6^. At this stage, copiotrophic flora such as *Actinobacteria*, *Firmicutes*, and *Proteobacteria* dominated in straw decomposition^7,8^, and β-*N*-acetylaminoglucosidase and leucine aminopeptidase also showed high activity and gradually decreased with the consumption of easily degradable substrates^9^. Difficult-to-decompose substances in straw (e.g., lignin, waxes, and tannins) gradually accumulated and were slowly decomposed by specific microorganisms in the late stages of straw decomposition^10,11^. At this stage, the abundance of oligotrophic *Acidobacteria*, *Chloroflexi*, and *Saccharibacteria* gradually increased^12,13^, and the activities of β-cellobiohydrolase and phenol oxidas showed high activity^14,15^. The decomposition of crop straw is a long-term process. Most of the existing studies are short-term decomposition experiments, with the majority of studies on decomposition of crop straw in the growing season^16,17^. During the non-growing season, the conversion of organic and inorganic components of straw into soil is still ongoing, and the composition of microbial communities is also changing. However, there is still a lack of research on characteristics of long-term straw decomposition and microbial community succession^18,19^.

Rice is a major grain crop in south China and produces more than 200 million tons of straw every year^1^. The input of straw nutrient resources has great potential for reducing fertilizer application. Sufficient soil water and higher temperature are beneficial to the straw decomposition and are not the limiting factors in the paddy soil^20^, and the rice straw decomposition may be mainly affected by biological factors. Therefore, it is of great significance to study the change in microbial abundance and community composition during the straw decomposition process to reveal the microbial mechanism of straw decomposition. Previous studies found that differences in crop straw components also lead to differences in characteristics of straw decomposition and microbial community succession^21^. Moreover, most of the previous experiments on straw decomposition in the field were conducted during the growing season of the crop^17^, and the characteristics of rice straw decomposition and the structure of the microbial community in long-term straw decomposition in the double-rice system are still unclear. Given this, A 2-year continuous experiment on rice straw decomposition was established in a double-cropping rice system in southeastern China. Our objectives were to (i) determine the dynamic of nutrient release and enzyme activity changes in straw and (ii) clarify the characteristics of succession in bacterial community composition and the factors influencing the succession during the straw decomposition.

## Results

### Changes in straw dry matter and nutrient release

**Figure 1:**
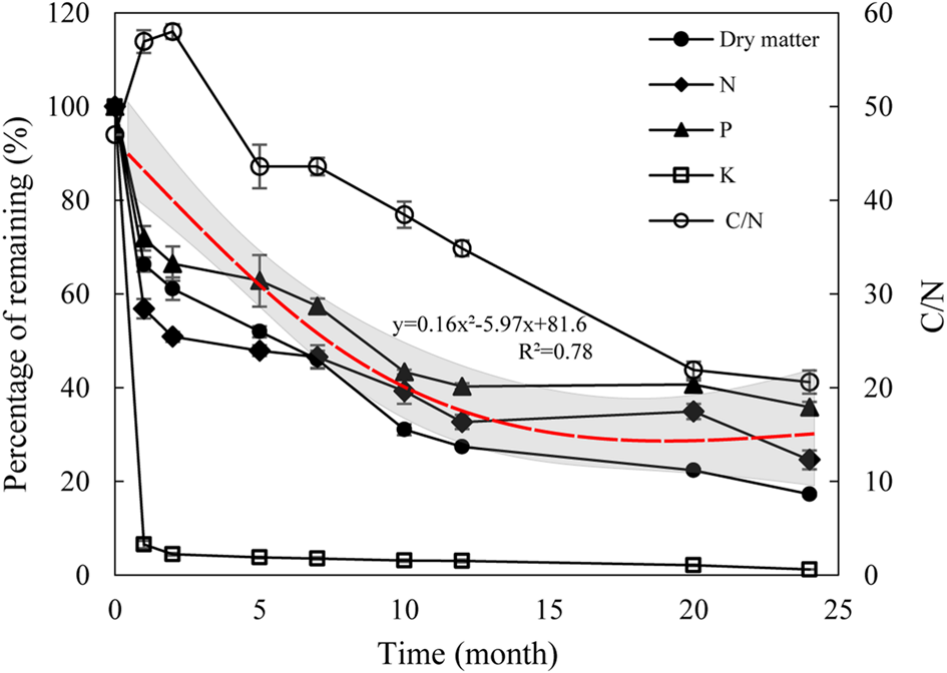
Dynamics of dry matter, N, P, K and C/N in straw decomposition.

The cumulative loss rate of straw dry matter mass was 33.7%, 38.9%, 48.1%, 53.9%, 69.0%, 72.6%, 77.6%, and 82.7% after 1, 2, 5, 7, 10, 12, 20, and 24 months since straw burial in soil (Fig. 1). The changes in the release rates of N and P from straw across the decomposition process were similar to the changes in dry matter loss rates, and they met the fitting formula of y = 0.16x^2^ − 5.97x + 81.6 (x = time, y = release rate of nutrients or dry matter from the straw, R^2^ = 0.78). 93.5% of the total straw K was released after 1 month. The straw C/N increased in the initial 2 months and gradually decreased with prolonged experimental time.

### Changes in straw enzyme activity and bacterial abundance

**Figure 2:**
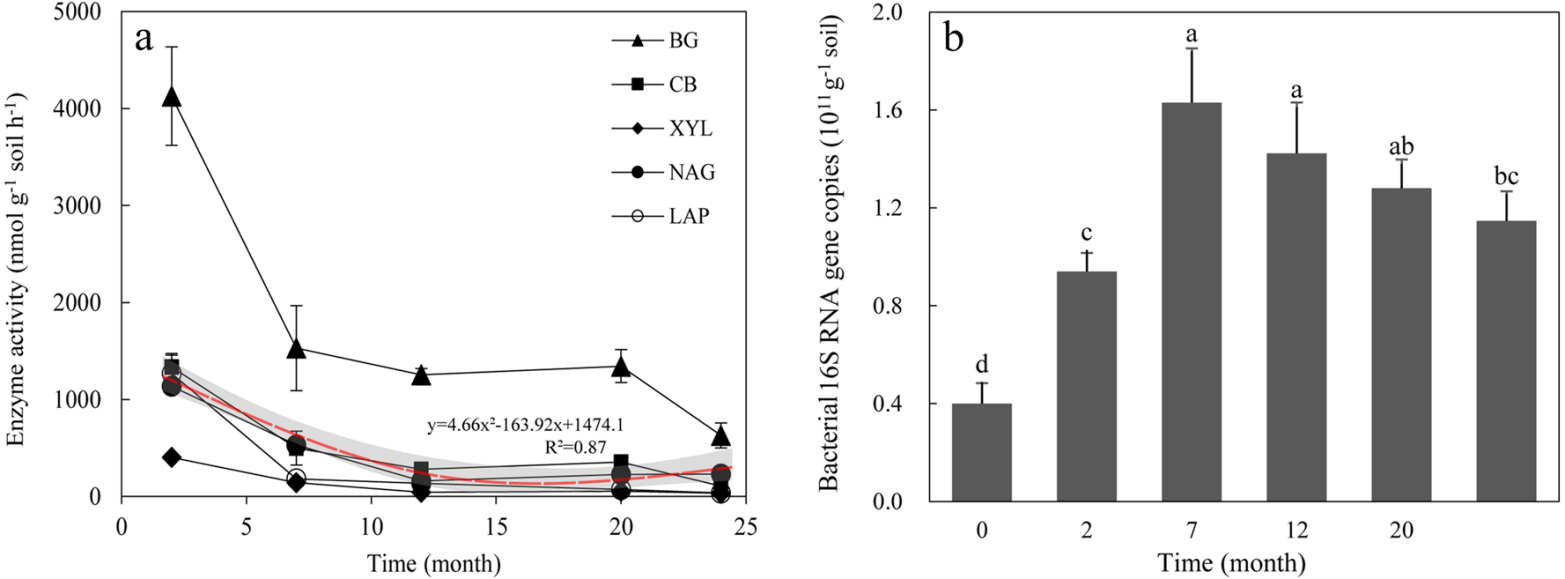
Changes in enzyme activity (**a**) and bacterial abundance (**b**) during straw decomposition. Different lower letters at the top of the columns present significant differences among decomposition periods (P < 0.05). BG, CB, XYL, NAG, and LAP are β-glucosidase, β-cellobiohydrolase, β-xylosidase, β-*N*-acetylglucosaminidase, and Leucine aminopeptidase activities, respectively.

The activities of BG, CB, XYL, NAG, and LAP were the highest in the initial stage of straw decomposition and then decreased significantly with test time. The changes in the activities of CB, NAG, and LAP with the straw test time can be explained by the fitting formula ‘y = 4.66x^2^ − 163.92x + 1474.1’ (x = time, y = enzyme activity, R^2^ = 0.87) (Fig. 2a). The copy number of bacterial 16S rRNA gene of straw gradually increased in the first 7 months after the straw was buried in soil, and then decreased with experimental enlarging time (Fig. 2b).

### The bacterial α-diversity during straw decomposition

The OTUs, ACE, Chao1 and Shannon index of straw bacteria increased significantly compared with those in the original straw (before burial in soil) and showed a trend of first increasing and then stabilizing with the passage of test time, and the maximum was all in the 12th month, but the Simpson index shows the opposite trend to the Shannon index (Table 1).

**Table 1:** α-diversity index of bacterial communities in straw decomposition.

Time (month)	OTUs	ACE	Chaol	Shannon	Simpson	Coverage
0	282.3c	291.5c	287.2c	3.0c	0.157a	0.998a
2	638.7b	1218.3b	883.2b	4.3b	0.036b	0.992a
7	2206.7a	2731.1a	2679.1a	6.1a	0.005c	0.982a
12	2468.0a	2960.2a	2885.1a	6.6a	0.005c	0.981a
20	2278.7a	2751.6a	2683.3a	6.3a	0.003c	0.982a
24	2339.7a	2806.5a	2742.2a	6.5a	0.003c	0.983a

The data represents the mean (*n* = 3). Different lower-case letters in one column indicate significant differences among treatments (P < 0.05).

### The bacterial β-diversity and influence of environmental factors on community composition during straw decomposition

**Figure 3:**
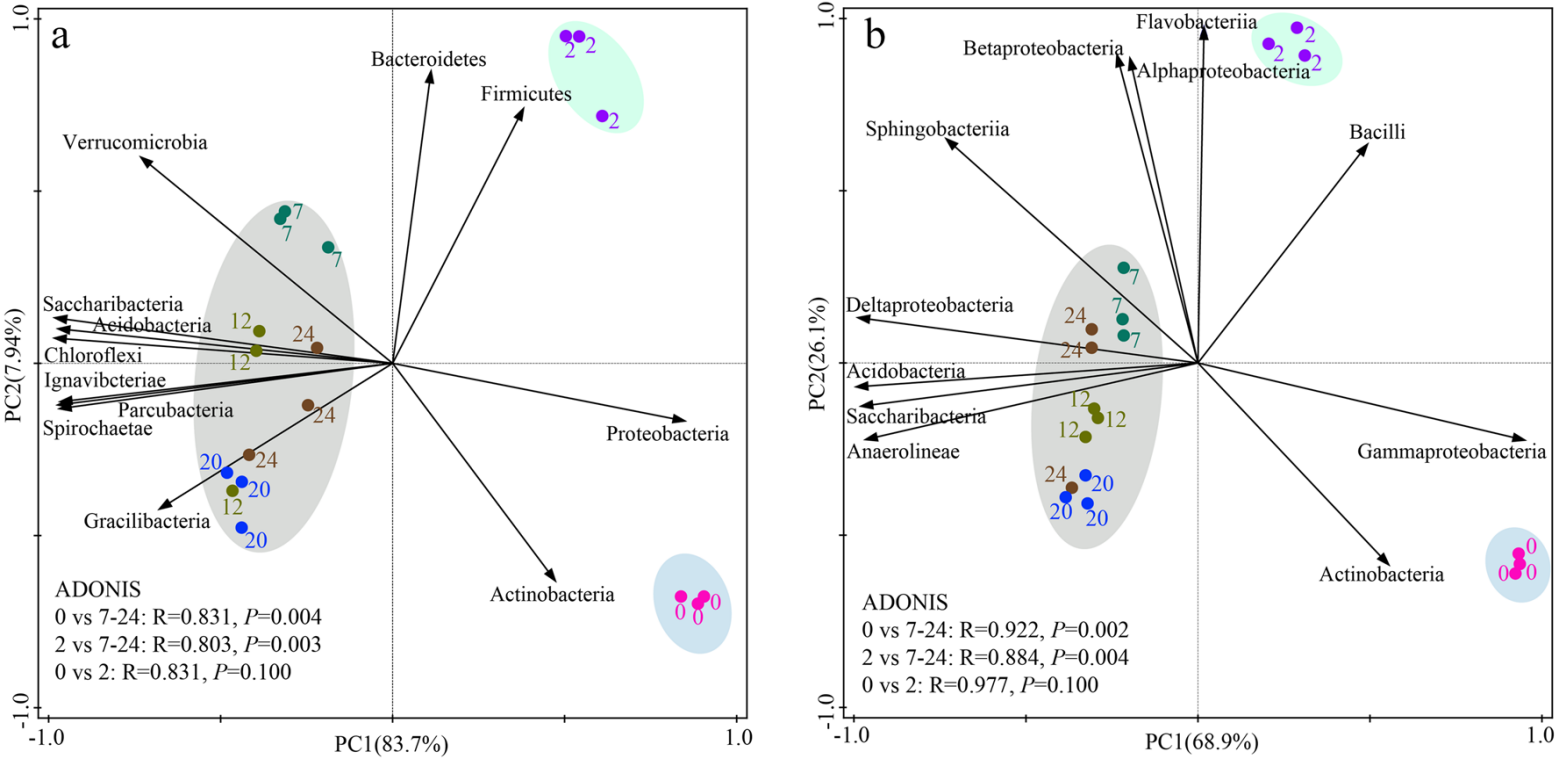
Principal Component analysis of straw bacterial community composition at phylum (**a**) and class (**b**) levels for different months of decomposition.

Principal component analysis showed that the bacterial phylum community composition in the 0 and 2nd months was significantly separated from other periods along the PC1 axis (Fig. 3a). Starting from the 0 month, the composition of the bacterial community is roughly distributed along the PC1 axis from the positive axis to the negative axis, and the community composition at 7–24 months did not separate significantly. The bacterial community in the initial straw was dominated by *Actinobacteria* and *Proteobacteria*, and *Bacteroidetes* and *Firmicutes* dominated the community in the 2nd month. The leading roles of *Acidobacteria*, *Chloroflexi*, *Ignavibacteriae*, *Gracilibacteria*, *Parcubacteria*, *Saccharibacteria*, *Spirochaetae*, and *Verrucomicrobia* increased significantly during the 7–24 months. For bacteria at the genus level, the bacterial community in the initial straw is dominated by *Actinobacteria* and *Gammaproteobacteria*; *Bacilli* and *Flavobacteriia* dominate the community composition in the 2nd month; *Acidobacteria*, Anaeroline*ae*, *Deltaproteobacteria*, *Saccharibacteria*, and *Sphingobacteriia* dominated the community composition during the 7–24 months of straw burial (Fig. 3b).

**Figure 4:**
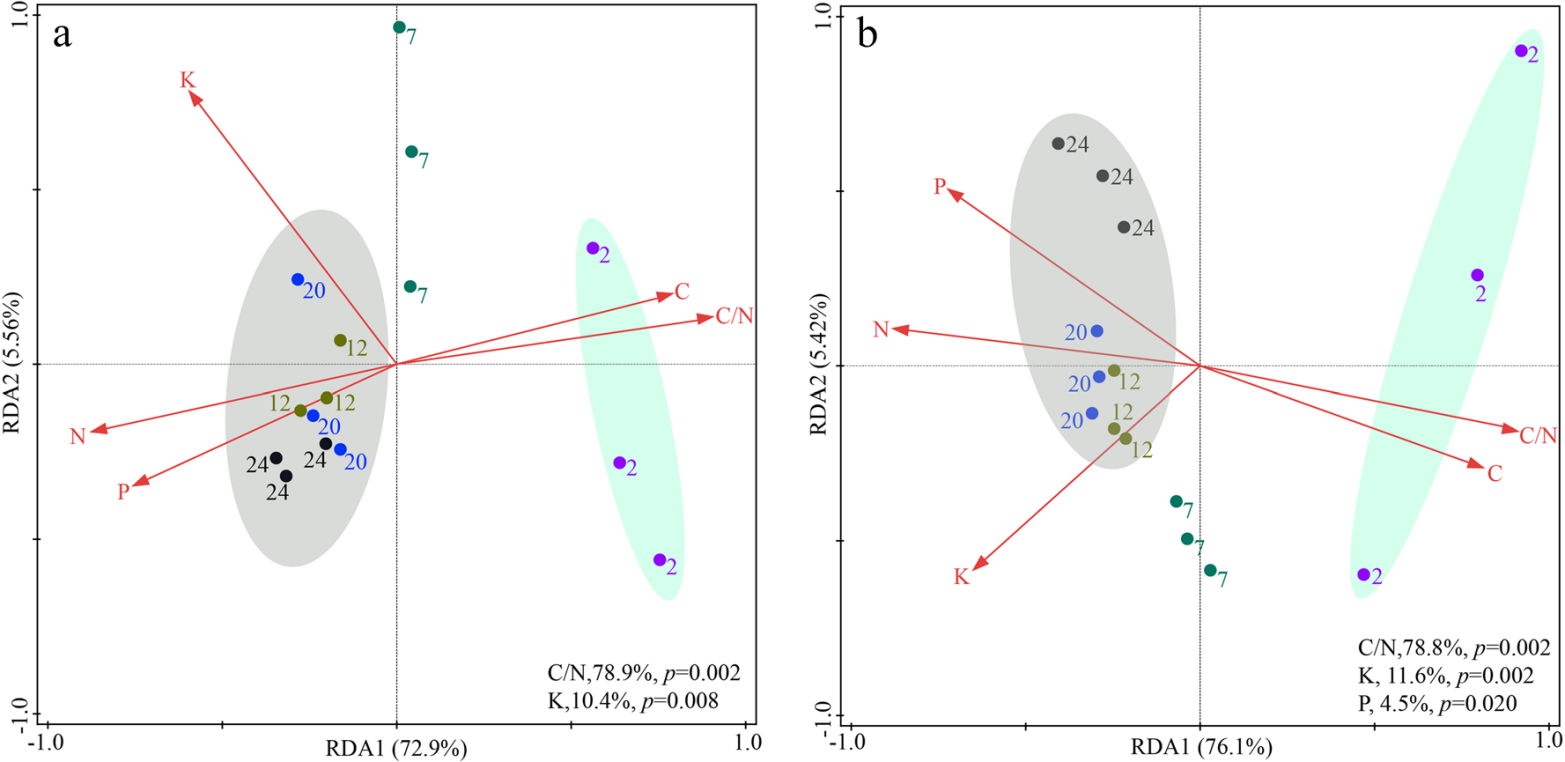
Redundancy analysis of straw bacterial community composition at phylum (**a**) and class (**b**) levels.

Redundancy analysis showed that bacterial community composition at the phylum level was mainly influenced by straw K and C/N, while bacterial community composition was mainly influenced by straw C/N, K and P at the class level (Fig. 4a,b).

### Succession of bacterial community composition during straw decomposition

**Figure 5:**
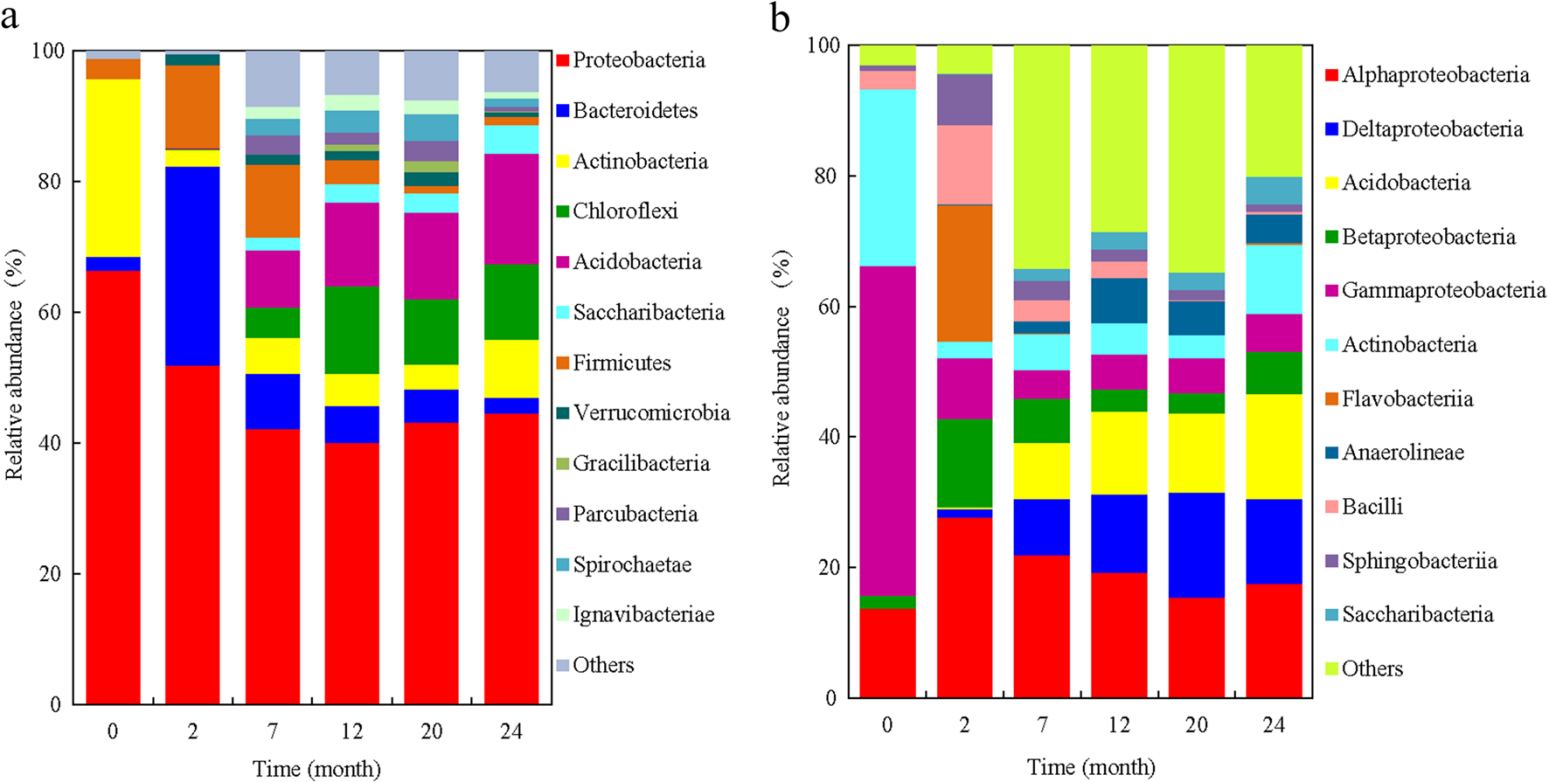
Changes in phylum (**a**) and class (**b**) levels of bacterial community composition during straw decomposition.

The relative abundance of phylum Proteobacteria during straw decomposition was the highest, accounting for 39.9‒52.0% of the total bacterial abundance of the sample, followed by *Bacteroidetes* (2.3‒30.3%), *Actinobacteria* (2.6‒8.7), *Chloroflexi* (0.1‒13.3%), *Acidobacteria* (0.2‒16.5%), *Saccharibacteria* (0.1‒4.4%), *Firmicutes* (1.0‒12.7%), *Verrucomicrobia* (0.7‒2.0%), *Gracilibacteria* (0.01‒1.5%), *Parcubacteria* (0.01‒2.9%), *Spirochaetae* (0.01‒3.7%) and *Ignavibacteriae* (0.01‒2.4%) (Fig. 5a). After the straw was buried in the soil, the relative abundance of *Proteobacteria* and *Actinobacteria* decreased gradually, and *Acidobacteria*, *Chloroflexi* and *Saccharibacteriamen* gradually increased with increasing experimental time, while *Bacteroides* and *Firmicutes* presented an increase–decrease change across the experiment process, and the highest abundance occurred in the 2nd month (Fig. S1a).

The bacterial class *Alphaproteobacteria* has the highest abundance, accounting for 15.4% to 27.7% of all components of the sample, followed by *Deltaproteobacteria* (1.3‒16.1%), *Acidobacteria* (0.2‒16.5%), *Betaproteobacteria* (3.1‒13.6%), *Gammaproteobacteria* (4.4‒9.3%), *Actinobacteria* (2.6‒10.9%), *Flavobacteriia* (0.01‒20.9%), *Anaerolineae* (0.01‒6.9%), *Bacilli* (0.1‒12.2%), *Sphingobacteriia* (1.2‒7.8%) and *Saccharibacteria* (0.08‒4.4%) (Fig. 5b). The abundance of *Acidobacteria* and *Saccharibacteria* gradually increased with the passage of test time, while the relative abundance of *Flavobacteria* and *Gammaproteobacteria* gradually decreased. The relative abundance of *Bacilli*, *Alphaproteobacteria*, *Betaproteobacteria*, and *Sphingobacteriia* showed an increasing–decreasing trend, which was the highest in the 2nd month. The relative abundance of *Anaerolineae* and *Deltaproteobacteria* first increased and then decreased, reaching the peak at the 12th and 20th months, respectively (Fig. S1b).

## Discussion

Climate condition is considered to be the decisive factor for the decomposition of organic materials^22,23^. In this study, the temperature (approximately 20 ℃) in the early stage of straw decomposition provided favorable conditions for the rapid decomposition of straw. The decomposition rate of rice straw in the early stage (the first 3 months) was higher than that in the late stage. Because the labile fractions were abundant and easily decomposed in the early stage, the decomposition rate of straw gradually slowed down with the accumulation of recalcitrant components in the later stage^24,25^. Moreover, straw decomposition rate showed significant differences at similar conditions (similar temperature and moisture) between the two experimental years, and the decomposition was slower at the later stage relative to that at the early stage, although the high bacterial abundance at the later stage in this study, suggesting that in paddy fields of Southeast China, the straw chemical composition is a more important factor influencing its decomposition compared to external environmental conditions.

The release rate of straw N and P was similar to the dry matter loss rate with the increasing test month (Fig. 1). Previous studies have shown that about 40% of the P in the straw is involved in the formation of the cell wall, and N mainly exists in the straw in the organic state with a high degree of cementation, which leads to a strong synchronization between the release of straw N and P and the loss of straw dry matter^16,18^. In this study, the release rate of straw K was higher than that of N and P in this study because K in straw can be directly released mainly in the form of K salt^26^. The N release rate of straw in the first two months was greater than the loss rate of dry matter (C), which may be the main reason for the increase in C/N at the early stages of straw decomposition.

The extracellular enzymes secreted by microorganisms are catalytic agents for straw decomposition, and it is beneficial to clarify the microbial driving mechanism in straw decomposition by analyzing their changes in the process of straw decomposition^27,28^. The activities of the five extracellular enzymes involved in the conversion of C and N were significantly higher in the early stage of decomposition (Fig. 2). This may be that sufficient straw C and N in the early stage promoted the growth and reproduction of microorganisms involved in the C and N cycles, and a large number of related microorganisms further induced an increase in the secretion of C and N cycle enzymes^29^. In addition, it was shown that there was a significant correlation between enzyme activity magnitude during straw decomposition and straw C/N ratio (P < 0.05), which further indicates that the enzyme activity secreted by microorganisms is strongly influenced by the straw C and N content (Fig. S1). In this study, β-glucosidase still maintained a higher enzyme activity in the late stage of decomposition, indicating that it plays a very important role in the late stage of straw decomposition, which is consistent with the results of previous studies on the changes of the enzyme activity of recalcitrant organic matter decomposition^14^. Microorganisms are the main decomposers of straw, and their abundance and community composition can affect the rate of straw decomposition^25^. In this study, bacterial abundance and α-diversity continued to increase when the rate of straw decomposition decreased, which may be caused by a decrease in bacterial abundance that lags behind the decrease in residue biomass^26,30^, and we suggest that as straw decomposers, soil microorganisms near the straw bag continue to colonize the straw after obtaining a large amount of carbon source, which is the main reason why bacterial α-diversity continues to increase even after the reduction of straw decomposition rate.

This study showed that the copiotrophic phylum *Bacteroidetes*, *Firmicutes*, and *Proteobacteria* dominated bacterial community composition at the initial stage of decomposition (Fig. 3), which is because the labile C fractions and other easily degradable components in the early stage of straw decomposition provide an easily accessible and rich source of energy for the growth and reproduction of these bacteria, thus promoting the increase of their populations^8,12^. The *Acidobacteria*, *Chloroflexi*, and *Saccharibacteria* gradually increased with the progress of the experiment, these three phyla were oligotrophic populations and dominated during the decomposition of residues with high recalcitrant fractions (e.g. lignin, cellulose) and poor nutrients in the later stage of residue decomposition^13^. *Bacilli* (soil saprophyte) was mainly found in the early stage of straw decomposition and consistent with the previous results^31^. The study found that the abundance of *Actinobacteria* increased in the late stage. Bernard et al. also reported the increase of *Actinobacteria* in the final stage of wheat straw degradation and believed that these oligotrophic bacteria might participate in the stimulating effect of straw decomposition^32^. The correlation analysis between the straw decomposition rate at different sampling periods and the relative abundance of different taxa showed that the rate of straw decomposition was significantly and positively correlated with the relative abundance of *Proteobacteria*, *Bacteroidetes*, *Alphaproteobacteria*, *Bacilli*, *Betaproteobacteria*, *Gammaproteobacteria*, *Sphingobacteriia*, and *Flavobacteriia* (Fig. S1), indicating that increasing the abundance of these taxa may facilitate the increase of straw decomposition rates. In addition, we also found that the changes in the relative abundance of different classes of bacteria within the same phylum level were inconsistent throughout the experiment (Fig.S2), implying that bacterial communities within the same phylum have functional differences at different stages of straw decomposition^13^.

The microorganisms colonizing crop residues mainly come from the soil near the residues^24,33^. However, the effect of soil biochemical properties near the straw bags on them was not explored in this experiment, which should be taken into account in future studies. In addition, fungi can effectively break down recalcitrant compounds within the straw by secreting residue degrading enzymes^15,34^. In future studies, we will explore the synergistic effects of fungal and bacterial communities during the decomposition of rice straw, which in turn will provide a reference for the isolation of microbial strains for rice straw degradation.

## Conclusions

The rate of rice straw decomposition and nutrient release was greater at the first 2 months after burial in soils, and gradually decreased with increasing experimental time in Southeast China. Rice straw decomposition rate showed a significant positive correlation with extracellular enzyme activity. Classes *Bacilli* and *Flavobacteriia* played a key role in the early stage of straw decomposition, while *Acidobacteria*, *Anaerolineae*, *Deltaproteobacteria*, *Saccharibacteria*, and *Sphingobacteria* dominated bacterial communities in the later stage. The change in straw chemical composition was the main factor affecting straw bacterial flora in Southeast China.

## Material and methods

### Experimental site

**Figure 6:**
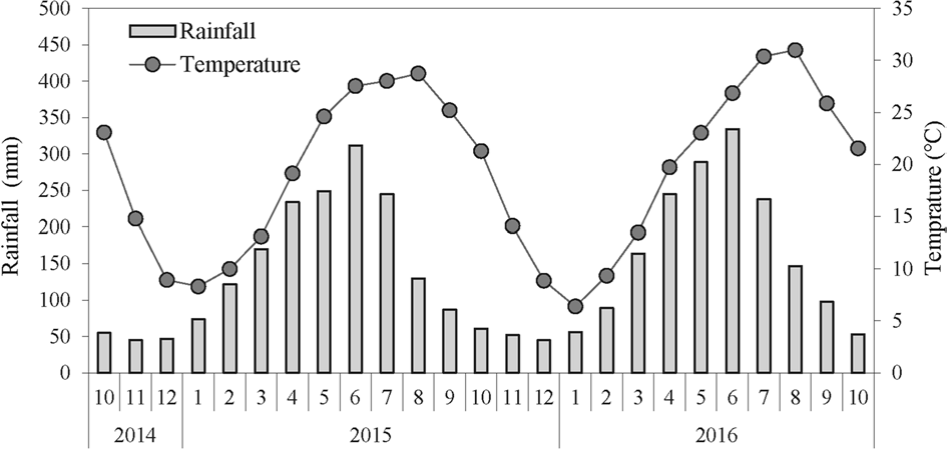
Rainfall and temperature of the experimental site during the experiment.

The straw decomposition experience was carried out at Nanchang Experimental Station of Jiangxi Academy of Agricultural Sciences (116°20'E, 28°15'N) from October 2014 to October 2016. The area has a subtropical monsoon climate with double-cropping rice as the main planting system. The annual average temperature and precipitation are 17.7 °C and 1374 mm, respectively. The physicochemical properties of the original soil (0–20 cm) before the start of the experiment: pH 6.6 (water: soil = 2.5:1), organic carbon 9.2 g kg^−1^, total nitrogen (N) 1.31 g kg^−1^, mineral N (nitrate N + ammonium N) 57.0 mg kg^−1^, available phosphorus (P) 13.6 mg kg^−1^, available potassium (K) 108.0 mg kg^−1^, the data of rainfall and temperature during the testing period were shown in Fig. 6.

## Experimental design

Rice straws (stems and leaves) were collected from the experimental field during the late rice harvest in October 2014. The straws were dried at 65 °C and cut into pieces (1–2 cm × 0.3–1 cm), and 12 g straw (8 t hm^−2^) was put into a nylon net bag (15 cm × 10 cm). Subsequently, 60 straw bags were embedded in the paddy soil (15 cm depth, 20 cm spacing distance). In order to avoid the influence of fertilizer on the determination of residual nutrients, no fertilizer was applied to the field during the experiment.

### Straw bag sampling

Six straw bags were collected at 0, 1, 2, 5, 7, 10, 12, 20, and 24 months after being buried, the soil particles and soil animals attached to the straw bags were removed, and the samples were transported to the laboratory immediately in the ice box. After three straw bags were dried to a constant weight at 65 ℃, the dry matter and nutrient content were measured. The other three straw bags at 0, 2, 7, 12, 20 and 24 months were stored at −70 °C for enzyme activity and bacterial community composition determination.

### Straw dry matter, nutrient and enzyme activity analysis

The straw sample was dried at 70 °C to a constant weight and then the dry matter was determined. The C and N of straw were determined by C/N element analyzer (Model CN, Vario Macro Elementar, Germany), P was determined by the H_2_SO_4_–H_2_O_2_ digestion-vanadium molybdenum yellow colorimetric method, K was measured by atomic absorption spectrophotometer (SpectAA-50/55, Varian, Australia)^35^. The cumulative loss rate of straw dry matter mass (A) and the release rate of straw nutrients (B) were calculated using the equation as following:

1 $$\mathrm{A}\left(\mathrm{\%}\right)=\frac{\left({W}_{1}-{W}_{2}\right) }{100\times {W}_{1} } $$

2 $$\mathrm{B}\left(\mathrm{\%}\right)=\frac{\left({W}_{1}{m}_{1}-{W}_{2}{m}_{2}\right) }{100\times {W}_{1}{m}_{1} } $$

*W*_1_ and *m*_1_ are the original dry matter weight of straw (g) and the original straw nutrient content (g kg^−1^); *W*_2_ and *m*_2_ are the dry matter weight (g) and nutrient content of straw after decomposing at different sampling times (g kg^−1^).

A fresh sample of straw equivalent to 0.3 g dry weight was weighed and placed in a centrifuge tube. The sample was thoroughly mixed with 50 ml sodium acetate buffer (50 mM) using a vortexer, and then the suspension was poured into a beaker, and a magnetic rotor was put in to maintain a uniform suspension. Buffer, sample suspension, 10 mM control and 200 mM substrate were sequentially added to a 96-well microtiter plate, incubated at 25 °C in the dark for 4 h, and then used with a microplate reader (Scientific Fluoroskan Ascent FL, ThermoFischer Scientific, Waltham, MA) for fluorescence quantification. The measurement conditions were excited at 365 nm, and the signal was recovered at 450 nm, followed by determination of β-glucosidase, β-cellobiohydrolase, β-xylosidase, β-*N*-acetylglucosaminidase, and Leucine aminopeptidase activities^36,37^. The enzyme activity unit is nmol g^−1^ h^−1^.

### Microbial total DNA extraction, PCR amplification, and Illumina NovaSeq sequencing

A 0.2 g straw sample was used for the microbial DNA extraction using the FastDNA^®^ SPIN kit (MP Biomedicals, Illkirch, France). The extracted DNA was purified using Ultra-Clean DNA cation kit (MoBio, Carlsbad, CA, USA) and was checked using 1.0% agarose gel electrophoresis. SYBR Green I was used to quantify the abundance of bacterial 16S rRNA gene copies in the iCycler system (USA) and the results were analyzed by Bio-Rad iQ5 v2.0. The PCR reaction system (20 μl) contains 2 × 10 Super Mix (Bio-Rad, USA), 10 μM primers (515F and 806R) and 1 μl of 1/10 diluted DNA. PCR was performed using the following thermal cycling program: 95 °C for 1 min, followed by 40 cycles at 94 °C for 15 s, 55 °C for 34 s, and 72 °C for 15 s. The plasmid containing bacterial DNA was diluted into different gradients to prepare a reaction standard curve to calculate the bacterial abundance. The V3-V4 region of the bacterial 16SrRNA gene was amplified by PCR using primers 338F (5'-ACTCCTACGGGAGGCAGCAG-3') and 806R (5'-GGACTACHVGGGTWTCTAAT-3') (95 °C for 2 min; 95 °C for 30 s, 56 °C for 30 s, 72 °C for 60 s, 30 cycles; 72 °C for 5 min final extension), the amplified product was recovered with 2% agarose gel, purified with AxyPrep DNA purification kit (Axygen Biosciences, Union City, CA, USA), and quantified with QuantiFluor ™-ST (Promega, USA). The purified PCR products were subjected to high-throughput sequencing using the Illumina MiSeq platform. Then the original sequence is subjected to quality control and processing using QIIME (V1.17). In order to avoid deviations caused by differences in sequencing depth, 33,480 sub-samples of each sample were randomly selected for read leveling and used for bacterial diversity and community composition analysis. UPARSE (V7.1) was used to classify OTUs according to 97% similarity, and UCHIME was used to identify and remove chimeric sequences. The Silva database was used to analyze the classification of each 16S rRNA gene sequence. Shannon, Simpson, Chao1 and ACE indices were calculated by Mothur software. The sequencing results were stored in the NCBI database (Accession number: PRJNA623251).

### Statistical analysis

The bacterial gene abundance, diversity index, and relative abundance at different sampling times were compared by one-way ANOVA based on Fisher's least significant difference (*P* < 0.05) using SPSS 19.0 (SPSS, Inc., Chicago, IL, USA). Canoco (V5.0, Ithaca, NY) was used for principal component analysis and redundancy analysis, and the R package "Vegan" was used for the ADONIS analysis (999 permutations) of bacterial communities during straw decomposition.

### Statement

All rice straw samples collected in this study have been licensed. All the rice straw experiments were in compliance with relevant institutional, national, and international guidelines and legislation.

## Supplementary Information

Supplementary Figures.

## Data Availability

The datasets generated during and/or analyzed during the current study are available from the corresponding author on reasonable request.
