# Supplementary material for: Characteristics of rice straw decomposition and bacterial community succession for 2 consecutive years in a paddy field in southeastern China
Source: Sci Rep. 2022 Dec 3;12:20893. doi: 10.1038/s41598-022-25229-8 (PMC9719457; doi:10.1038/s41598-022-25229-8)
Supplement: Supplementary file 1 — Supplementary Figures. [file 41598_2022_25229_MOESM1_ESM.docx]

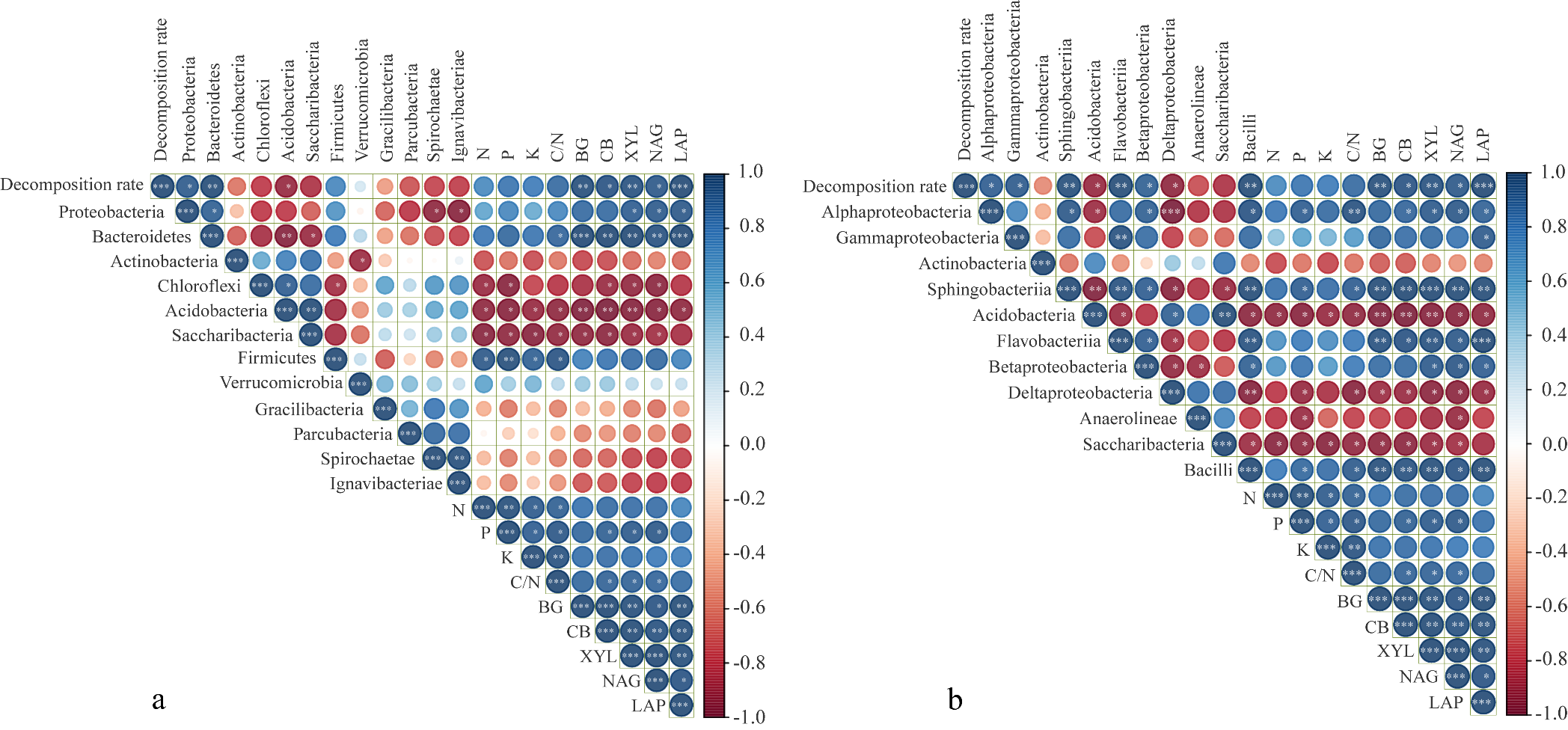


**Figure.S1** Correlation analysis between straw decomposition rate, bacterial community composition (a: phylum, b: class) and enzyme activity during rice straw decomposition.

The symbol “*” indicates significant correlation between items (P<0.05), “**” represents a highly significant correlation between items(P<0.01), and “***” represents a highly significant correlation between items (P<0.001).

a

**Figure.S2** Changes in the relative abundance of bacterial dominant phyla (a) and classes (b) (OTUs with relative abundance more than 1%) in straw decomposition. Bars are standard errors (n=3). Different letters indicate significant differences among sampling times (P<0.05*).*
